# ATO/ATRA/Anthracycline-Chemotherapy Sequential Consolidation Achieves Long-Term Efficacy in Primary Acute Promyelocytic Leukemia

**DOI:** 10.1371/journal.pone.0104610

**Published:** 2014-08-14

**Authors:** Zi-Jie Long, Yuan Hu, Xu-Dong Li, Yi He, Ruo-Zhi Xiao, Zhi-Gang Fang, Dong-Ning Wang, Jia-Jun Liu, Jin-Song Yan, Ren-Wei Huang, Dong-Jun Lin, Quentin Liu

**Affiliations:** 1 Department of Hematology, Third Affiliated Hospital, Sun Yat-sen University, Sun Yat-sen Institute of Hematology, Sun Yat-sen University, Guangzhou, China; 2 Institute of Cancer Stem Cell, Dalian Medical University, Dalian, China; 3 Department of Hematology, Second Affiliated Hospital, Dalian Medical University, Dalian, China; European Institute of Oncology, Italy

## Abstract

The combination of all-trans retinoic acid (ATRA) and arsenic trioxide (As_2_O_3_, ATO) has been effective in obtaining high clinical complete remission (CR) rates in acute promyelocytic leukemia (APL), but the long-term efficacy and safety among newly diagnosed APL patients are unclear. In this retrospective study, total 45 newly diagnosed APL patients received ATRA/chemotherapy combination regimen to induce remission. Among them, 43 patients (95.6%) achieved complete remission (CR) after induction therapy, followed by ATO/ATRA/anthracycline-based chemotherapy sequential consolidation treatment with a median follow-up of 55 months. In these patients, the estimated overall survival (OS) and the relapse-free survival (RFS) were 94.4%±3.9% and 94.6±3.7%, respectively. The toxicity profile was mild and reversible. No secondary carcinoma was observed. These results demonstrated the high efficacy and minimal toxicity of ATO/ATRA/anthracycline-based chemotherapy sequential consolidation treatment for newly diagnosed APL in long-term follow-up, suggesting a potential frontline therapy for APL.

## Introduction

Acute promyelocytic leukemia (APL), characterized by the t (15, 17) chromosomal translocation and leukemogenic PML-RARα fusion protein, is accumulated of abnormal promyelocytes in the bone marrow and causes severe bleeding tendency [1]. The treatment of APL with chemotherapy achieved complete remission (CR) in two-thirds of newly diagnosed patients, however, the 5-year disease-free survival (DFS) was still very poor [1]–[3]. The induction of all-trans retinoic acid (ATRA) in the treatment and optimization of the anthracycline-based regimens resulted in terminal differentiation of APL cells with a 90–95% CR and the 5-year DFS up to 74% [1], [4], [5], although approximately 5–30% of patients developed disease recurrence [6].

As one of the most potential drugs in APL, arsenic trioxide (As_2_O_3_, ATO) targets PML/RARα and exerts dose-dependent dual effects on APL cells, with low concentrations inducing differentiation and high concentrations triggering apoptosis [7]. Since 1990s, the use of ATO has improved the clinical benefit of refractory or relapsed as well as newly diagnosed APL [8]–[11]. ATO injection for APL patients who developed disease recurrence or failed to respond to standard treatment was later approved by the US FDA. Moreover, molecular remission is obtainable in patients from 72% to 91% after CR by ATO alone [12], [13]. Strong synergistic anti-leukemic effects of ATO in combination with ATRA were found in both APL cell lines and APL animal models, with induction catabolism of the PML-RARα fusion protein [14]–[17]. Importantly, previous clinical trials showed that the combination of ATO and ATRA yielded a longer survival rate compared to either ATRA or ATO monotherapy [18]–[23]. Moreover, ATO consolidation therapy spared anthracycline exposure [24], and improved both event-free survival (EFS) and overall survival (OS) in newly diagnosed APL [25]. Yet, a standard ATO/ATRA consolidation regimen for newly diagnosed APL remains to be further validated.

In this retrospective study, ATRA/chemotherapy combination regimen was applied to induce remission for newly diagnosed APL patients. A regimen consisting of ATO, ATRA and anthracycline-based chemotherapy was used sequentially as consolidation therapy for the patients who obtained CR. The long-term efficacy and safety of ATO/ATRA/anthracycline-based chemotherapy consolidation regimen were evaluated.

## Methods

### Patients

This retrospective study consisted of 45 patients with newly diagnosed APL in the Third Affiliated Hospital, Sun Yat-sen University, from March 1, 2000 to August 31, 2012. The median age was 29 years (10–62 years). Pertinent patient clinical reports of this study were obtained with patients' written consent and the approval of the Ethical Board of The Third Affiliated Hospital, Sun Yat-sen University ([2013]2-69). Parental written consent was obtained for underage participants.

**Figure 1:**
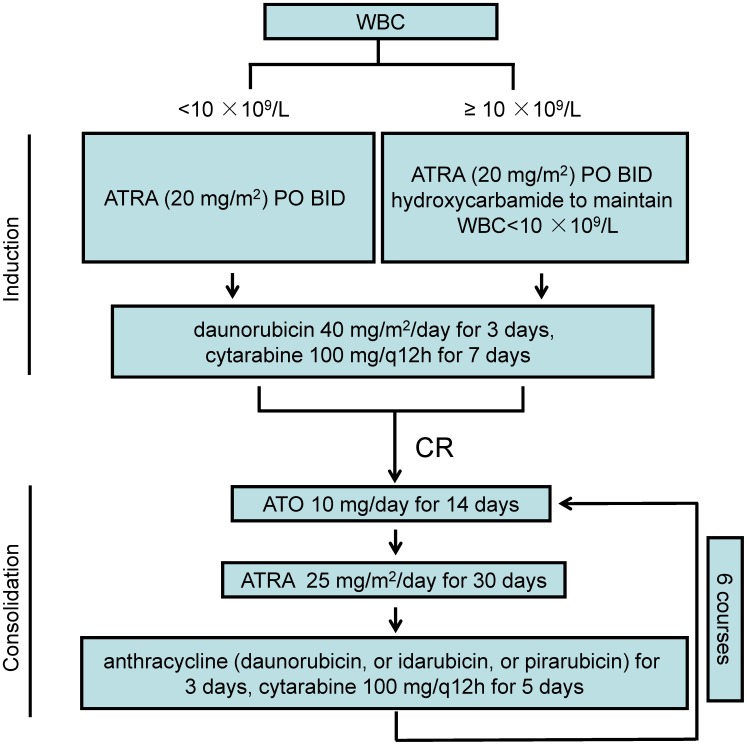
A chart review of patients treated with standard of induction and consolidation therapy.

APL diagnosis was established according to clinical presentations, morphological criteria of the French-American-British classification, cytogenetic assay for t (15; 17) (q22; q21) and RT-PCR analysis for PML-RARα transcripts. The exclusion criteria for this retrospective study included: dysfunction of liver or kidney; any heart diseases or cardiac functional insufficiency; patients who died before initiation of the therapy. Standard induction therapy was administered for the 45 newly diagnosed APL patients (Figure 1). Two patients died during induction treatment. The remaining 43 patients received consolidation therapy. The clinical features of patients were described in Table 1.

**Table 1:** Clinical data of the patients.

	N = 45
Gender, male/female	20/25
Median age, years	29 (10–62)
WBC, ×109/L	
Median	2.3 (0.2–47.5)
<10	1.9 (0.2–7.8, 84.4%)
≥10	37.9 (13.2–47.5, 15.6%)
Median Hb, g/L	81.0 (38.0–120.0)
Median platelet, ×109/L	23.0 (5.0–120.0)
Clinical CR	95.6%
Median days to clinical CR	30 (20–60)
Median months to molecular CR	6 (2–12)

### Remission Induction Therapy

Induction therapy for these newly diagnosed patients with APL was a combination of ATRA and daunorubicin plus cytarabine. Once the diagnosis was suspected on the basis of clinical features and the peripheral blood smear, ATRA was administered orally at 40 mg/m^2^/day (divided into two equal doses) until CR was achieved. Patients with WBC counts ≥10×10^9^/L additionally received hydroxycarbamide orally until the WBC count was down to less than 10×10^9^/L. ATRA was continued for 3 to 15 days to ameliorate the coagulopathy before initiating chemotherapy (daunorubicin 40 mg/m^2^/day for 3 days, cytarabine 100 mg/q12 h for 7 days).

### Supportive Care

During induction of remission, examinations including whole peripheral blood cell counts, renal and hepatic function tests were performed. Coagulation and fibrinolysis parameters including fibrinogen, D-dimmers, fibrin degradation product (FDP), prothrombin time (PT), and activated partial thromboplastin time (APTT) were monitored to identify the requirement of platelet, fresh plasma, or cryoprecipitate transfusions. Supportive treatment was based on maintaining platelet counts >30×10^9^/L until coagulopathy disappearance. Electrocardiogram and sonography were used for monitoring the cardiac function for patients. APL differentiation syndrome (APLDS) was treated with prednisone or dexamethasone until clear resolution of symptoms. Drug toxicities were documented using the National Cancer Institute-Common Toxicity Criteria, version 3.0. Symptomatic therapy was performed for the side effects of ATO, ATRA and anthracycline. Patients with chronic hepatitis B were treated with lamivudine or telbivudine for prevention of virus activation.

### Consolidation Therapy

Patients were monitored to confirm that the bone marrow morphology and recovery of peripheral blood cell counts. Consolidation therapy included 6 courses was initiated once CR was achieved, and each course included three consecutive regimens: (1) ATO, 10 mg/day for 14 days intravenously; (2) ATRA, 25 mg/m^2^/day for 30 days orally; (3) anthracycline-based regimens: daunorubicin (40 mg/m^2^/day), or idarubicin (8 mg/m^2^/day), or pirarubicin (25 mg/m^2^/day) for 3 days plus cytarabine 100 mg/q12 h for 5 days. The three regimens of consolidation therapy were administered sequentially every month in the first year after achieving CR. In the second year, each regimen of consolidation therapy was administered sequentially every two months. Six courses were given totally.

All patients received intrathecal therapy (methotrexate 15 mg, cytarabine 50 mg, dexamethasone 8 mg) when CR was achieved. Prophylaxis was performed 4–6 times altogether.

### Response Definition

CR was defined according to clinical presentations and morphological criteria, including cellular bone marrow blasts and abnormal promyelocytes≤5% with an absolute neutrophil count ≥1.0×10^9^/L and platelet count ≥100×10^9^/L. Clinical recurrence was defined as the presence of ≥5% blasts, or abnormal promyelocytes in the bone marrow, or the appearance of leukemic cells in peripheral blood, or abnormal promyelocytes in cerebrospinal fluid (CSF). RT-PCR for the PML-RARα fusion transcript was performed on the bone marrow follow-up every 2 months for monitoring molecular remission. After molecular remission, the examination was still performed every 3 months for monitoring relapse.

### Statistical Analysis

OS was defined as the time from the initiation of induction therapy to death. RFS was defined as the time from CR to relapse. Survival analysis was performed using Kaplan-Meier estimate methods. Statistical analysis was performed using SPSS16.0 for windows software.

## Results

### Outcomes

As seen in Table 1, among total 45 patients, 43 (95.6%) achieved CR in remission introduction therapy. The median time to achieve CR was 30 days (range: 20–60 days). Two patients suffered from early death within 15 days during the induction therapy due to intracranial hemorrhage (1 case), or acute tumor lysis syndrome (1 case). For the 43 patients who entered CR, all received ATO/ATRA/anthracycline-based chemotherapy for consolidation therapy. The median follow-up was 55 months (range: 6–150 months), and the median months to molecular CR was 6 months (range: 2–12 months). Till the end of this study, 41 patients remained in good clinical and molecular remission. Two patients relapsed: one presented with central nervous system (CNS) leukemia in the 27th month and the other developed full bone marrow relapse in the 10th month. Both patients died 6 months after relapse. No patient developed a secondary myelodysplastic syndrome or carcinoma.

**Figure 2:**
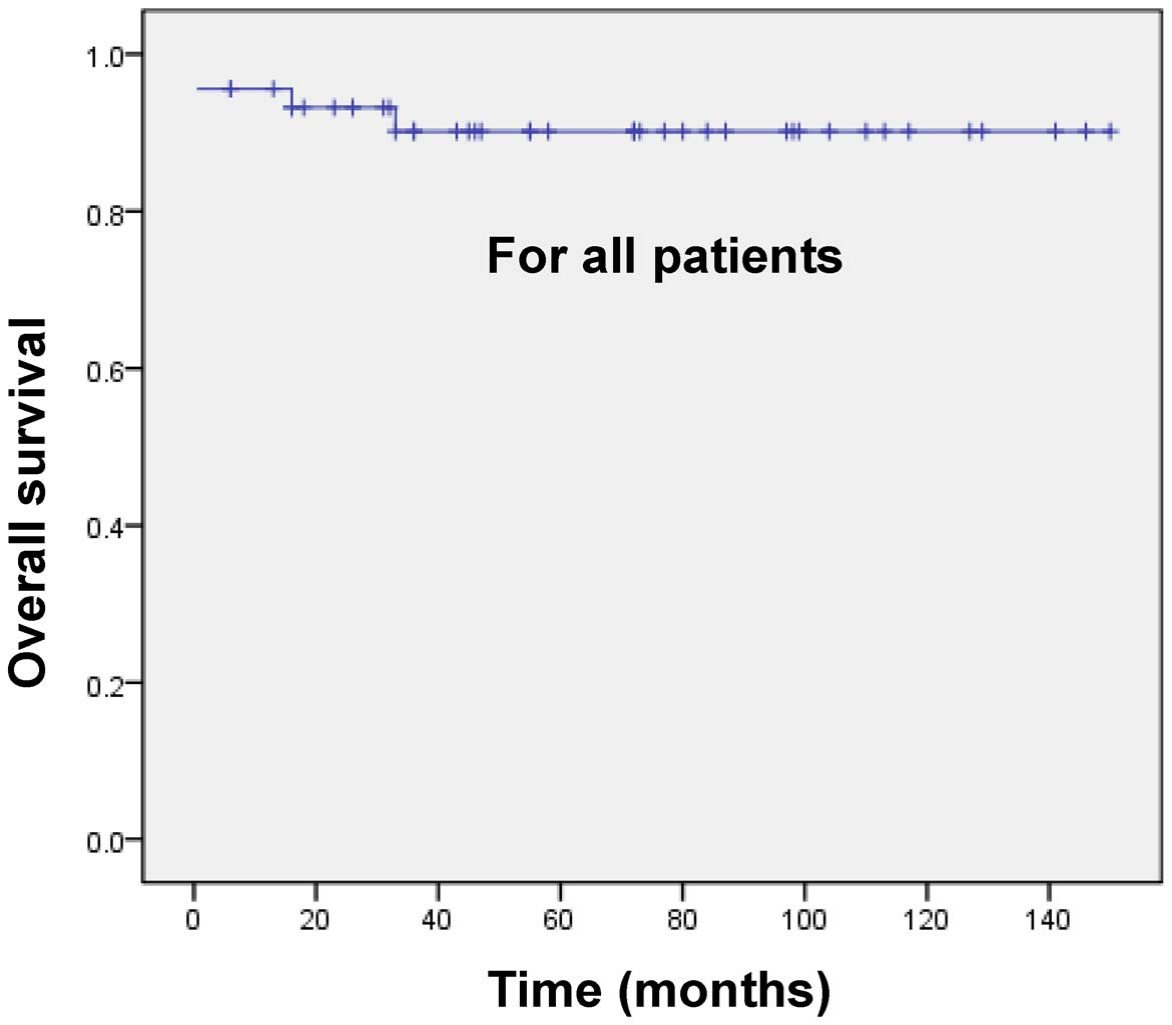
Survival analysis. The OS for all 45 patients.

**Figure 3:**
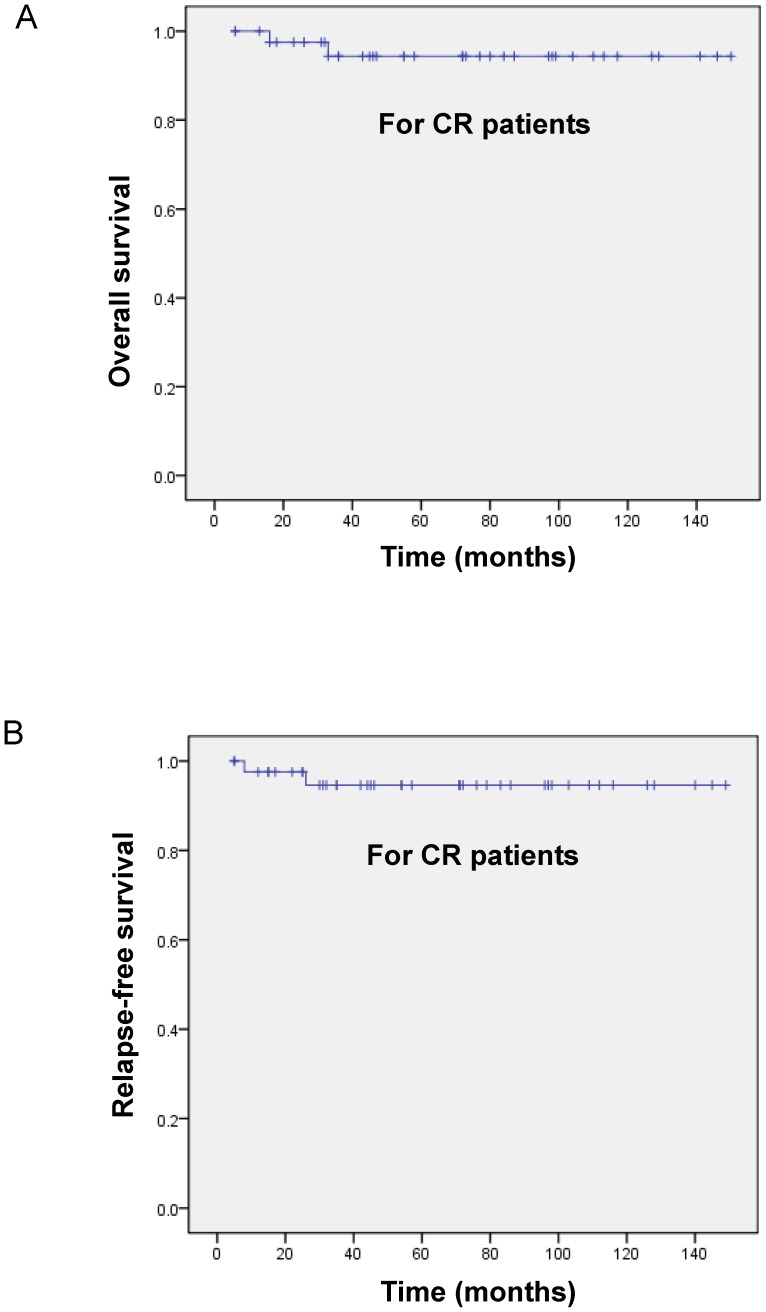
Survival analysis. The OS (A) and RFS (B) for the 43 patients who obtained CR.

As shown in Figure 2 and Figure 3, the estimated 3-year OS rates for all 45 patients and for those who achieved CR (n = 43) were 90.2%±4.7% (Figure 2) and 94.4%±3.9% (Figure 3A), respectively. The RFS rates for CR patients were 94.6±3.7% (Figure 3B).

### Toxicity Profile

The main side effect of ATRA is the APLDS whereas that of ATO is liver dysfunction. Both ATRA- and ATO- based treatments were tolerated well in the present study. As shown in Table 2, APLDS was diagnosed in 2 patients (4.7%). Other side effects of ATRA, such as skin reactions (19 patients, 44.2%), headache (13 patients, 30.2%), gastrointestinal tract reactions (6 patients, 14.0%) and fever (4 patients, 9.3%), were mild and overcame by administration of symptomatic medication. During consolidation, 6 of 41 patients developed tolerable and reversible grade I liver dysfunction and 1 patients developed grade II liver dysfunction, whereas no grade III–IV liver toxicity was observed. Hepatic function returned to normal in all of these patients after supportive therapy. No one needed termination of ATO therapy because of severe liver damage. Therapy-related neutropenia were observed in 8 patients (18.6%). One 62-year-old patient presented with chronic cardiac insufficiency in the 18th month after CR, which might be due to the accumulation of anthracycline for the elderly. In addition, all the 8 hepatitis B patients did not show any virus reactivation during consolidation.

**Table 2:** Toxicity profile.

	N = 43
Hepatotoxicity	7 (16.3%)
Grade I	6 (14.0%)
Grade II	1 (2.3%)
Grade III	0 (0%)
Grade IV	0 (0%)
Skin reaction	19 (44.2%)
Headache	13 (30.2%)
Neutropenia	8 (18.6%)
Gastrointestinal reaction	6 (14.0%)
Cardiac arrhythmia	1 (2.3%)
APLDS	2 (4.7%)
Fever	4 (9.3%)

## Discussion

ATRA in combination with anthracycline-based chemotherapy is considered as the standard for the induction and consolidation therapy of newly diagnosed APL. However, cumulative incidence of relapse still occurs in one third of the patients who have obtained CR. ATO induced catabolism of the PML-RARα fusion protein, demonstrating an effective targeted therapy in APL. In 1990s, the possibility of using a triad of chemotherapy, ATRA, and ATO for newly diagnosed patients in APL was discussed at a meeting in Shanghai. Then studies in the mouse model showed that this combination could dramatically prolong the survival or even eradicate disease. These results encouraged physicians to conduct new therapeutic approaches based on ATO/ATRA/anthracycline-based chemotherapy combination for the treatment of newly diagnosed APL patients.

Indeed, since the introduction of ATRA/ATO-based combination treatment for newly diagnosed APL and recurrence, the CR rate and the 5-year DFS have been greatly improved [18]–[23], [26]. In this study, the ATRA/chemotherapy combination regimen was administered to induce remission, and the ATO plus ATRA and anthracycline-based chemotherapy consolidation regimen was used to maintain long-term efficacy for newly diagnosed APL patients. In 45 *de novo* patients, CR was achieved in 43 patients (95.6%), whereas the median time to achieved CR was 30 days. The estimated 3-year OS rate for all patients was 90.2%±4.7%. For patients who achieved CR (n = 43), the OS and RFS rates were 94.4%±3.9% and 94.6±3.7%, respectively. Our data were consistent with recent studies [23], which reported a long-term outcome in the ATRA/ATO-based regimen.

The therapeutic benefit of ATO as a single agent for the treatment of APL has been reported previously [27], [28], thus using ATO as the post-remission therapy for the APL patients in CR was reasonable. Importantly, ATO consolidation produced a good survival rate no matter which method was used in CR induction and eliminated the need for maintenance therapy [29]–[31]. However, the relatively high incidence of ATO-induced hepatotoxicity during remission induction remains unclear and worthy of note, though the side effects of ATO were considered to be moderate. Reversible grade III–IV hepatotoxicity was seen in a small proportion of patients [27]. Overtreatment in the majority of patients was potentially associated with a risk of treatment-related death during early disease remission as well as longer-term risks of secondary carcinoma or anthracycline-related cardiomyopathy. Thus in the present study, ATRA-based induction regimen was applied and ATO was not added to the remission regimen. Either the daily or the total dosage of ATO for consolidation was minimal (10 mg/day for 14 days each course), which APL patients could benefit from ATO by consolidation without overtreatment during each course. In fact, during the consolidation, no grade III–IV hepatotoxicity was documented in our patients. Only 7 patients developed tolerable and reversible grade I–II liver dysfunction, and their hepatic function returned to normal after consolidation therapy. Other side effects were minimal during post-remission treatment. Another major concern associated with long-term exposure to ATO is secondary tumors, and we found no cases in the present study developed secondary tumors. Besides, our analysis showed that incorporation of ATRA drastically achieved long-term efficacy. Importantly, patients in our study showed a very low incidence of APLDS (4.7%). While the dosage of ATO was relatively small, APL patients could benefit from the consolidation with ATO and ATRA, thus usage of ATO/ATRA combination as the post-remission therapy for the APL patients in CR contributed to high efficacy and low side effects.

The therapeutic benefit of ATRA/ATO use in relapsed APL has been described previously [32]–[35]. However, in a randomized study of 10 cases, the ATRA/ATO combination regimen failed to induce synergistic effect [36]. In our study, the beneficial effects were observed in the newly diagnosed APL, in contrast to that report. The reason might be that majority of the relapsed patients lost sensitivity to ATRA due to previous exposure, making it difficult to expect a full efficacy of the synergism between ATRA and ATO in those patients. In addition, parts of recent studies about different risks of patients were summarized in Table 3 [23], [25], [31], [37]–[42] to make a comparison and we found that there was no strong evidence about the recommended strategy for different risk groups. However, the addition of ATO was proved to improve the long-term survival of patients with different risks, which gave support to our present study.

**Table 3:** Review of clinical studies of APL in different groups.

Clinical Studies	No. of patients	Age (median)	Sanz Risk (low/int/high)	Induction Therapy	CR	Consolidation Therapy	Maintenance Therapy	Survival Outcome
Long ZJ, et al. present study	45 (20/25)	29 (10–62)	low/int 38; high 7	ATRA+DNR+Ara-C	95.6%	ATO+ATRA+IDA+Ara-C, 6 courses		3-year OS 90.2%, RFS 94.6%
Zhang YM, et al. 2013 [37]	33 (18/15)	65 (60–79)	6/22/5	ATO	87.9%	ATO, 4 years		10-year OS 69.3%, DFS 64.8%, CSS 84.8%
Lo-Coco F, et al. 2013 [38]	A: 77 (40/37); B: 79 (36/43)	A: 44.6 (19.1–70.2); B: 46.6 (18.7–70.2)	A: low/int 33/44; B: low/int 27/52	A: ATRA+ATO; B: ATRA+IDA	A: 100%; B: 95%	A: ATO+ATRA, 28 weeks; B: ATRA+IDA/MTZ, 3 cycles	B: MTX, 6-MP, ATRA, 2 years	A: 2-year OS 99%, DFS 97%; B: 2-year OS 91%, DFS 90%
Iland HJ, et al. 2012 (APML4) [39]	124 (62/62)	44 (3–78)	32/67/24	ATRA+IDA+ATO	95%	ATO+ATRA, 2 cycles	ATRA, MTX, 6-MP, 8 cycles	2-year RFS 97.5%, FFS 88.1%, OS 93.2%
Avvisati G, et al. 2011 (AIDA 0493) [40]	828 (438/390)	37.2 (1.4–74.7)	157/432/231	ATRA+IDA	94.3%	IDA+Ara-C, MTZ+VP-16, IDA+Ara-C+6-TG, 3 courses	6-MP, MTX, ATRA, 2 years	12-year EFS 68.9%, OS 76.5%, DFS 70.8%
Sanz MA, et al. 2010 (LPA2005) [41]	402 (209/193)	42 (3–83)	84/200/118	ATRA+IDA	99%/95%/83%	IDA, ATRA, MTZ, Ara-C, 3 courses	6-MP, MTX, ATRA, 2 years	4-year DFS 90% (93%/92%/82%), OS 88% (96%/91%/79%)
Powell BL, et al. 2010 (C9710) [25]	A: 244 (123/121); B: 237 (124/113)	15–60 year 207/197; >60 year 37/40	A: 69/120/55; B: 67/112/58	A: ATRA+Ara-C+DNR; B: ATRA+Ara-C+DNR	A: 90%; B: 90%	A: ATO, 2 cycles+(ATRA+DNR), 2 cycles; B: (ATRA+DNR), 2 cycles	ATRA±6-MP/MTX, 1 year	3-year EFS 80%/63%, OS 86%/81%, DFS 90%/70%
Hu J, et al. 2009 [23]	85 (47/38)	>55 year 14; ≤55 year 71	low/int 66; high 19	ATRA+ATO	94.1%	DNR+Ara-C, Ara-C pulse, HHT+Ara-C, 3 cycles	ATRA, ATO, MTX/6-MP, 5 cycles	5-year OS 91.7%, RFS 94.8%.
Lengfelder E, et al. 2009 (AMLCG) [42]	142 (59/83)	40 (16–60)	33/72/37	ATRA+TAD (6-TG, Ara-C, DNR)+HAM (Ara-C, MTZ)	low/int 95.2%; high 83.8%	TAD, 1 cycle	Ara-C, DNR, 6-TG, CTX, 3 years	6-year EFS 78.3%/67.3%, OS 84.4%/73.0%, RFS 82.1%/80.0%
Asou N et al. 2007 (APL97) [31]	283 (158/125)	48 (15–70)	low/int 232; high 51	ATRA±IDA/Ara-C	94%	MTZ+Ara-C, DNR+VP-16+Ara-C, IDA+Ara-C, 3 courses	BHAC, DNR, 6-MP, MTZ, VP-16, VDS, ACR, 6 courses	6-year DFS 68.5%, OS 83.9%

Abbreviations: low/int/high: low/intermediate/high; OS: overall survival; DFS: disease-free survival; CSS: cause-specific survival; FFS: failure-free survival; Ara-C: cytarabine; BHAC: behenoyl Ara-C; DNR: daunorubicin; IDA: idarubicin; MTX: methotrexate; 6-MP: mercaptopurine; MTZ: mitoxantrone; VP-16: etoposide; VDS: vindesin; ACR: aclarubicin; 6-TG: 6-thioguanine; HHT: homoharringtonine; CTX: cyclophosphamide.

Mechanically, ATRA and ATO targets PML/RARα and exerts dose-dependent differentiation and apoptosis. Microarray, proteomics, and bioinformatics revealed that synergistic effect in combination therapy was due to transcriptional remodeling induced by ATRA-induced differentiation and ATO-related proteome level change. Importantly, enhanced degradation of PML-RARα might be considered for the efficacy of combination therapy in patients: ATO targeted PML, while ATRA aimed to RARα. Besides RA signaling and ubiquitin-proteasome pathway, some self-renewal and differentiation related molecules were newly revealed to be involved in the ATO/ATRA synergistic effect, such as c-myc, Bmi-1 [14], [43]. Thus, further studies should attempt to identify the network by which ATO/ATRA regulates in APL cells.

In summary, we reported that the ATO/ATRA-based regimen incorporating chemotherapy for consolidation therapy for newly diagnosed APL yielded an encouraging long-term survival rate with alleviated side effects, thus reinforcing its potential use as frontline therapy for APL.
